# A Transcriptomic Insight into the Impact of Colon Cancer Cells on Mast Cells

**DOI:** 10.3390/ijms20071689

**Published:** 2019-04-04

**Authors:** Yingxin Yu, Bart R. Blokhuis, Johan Garssen, Frank A. Redegeld

**Affiliations:** 1 Division of Pharmacology, Utrecht Institute for Pharmaceutical Sciences, Faculty of Science, Utrecht University, 3584CG Utrecht, The Netherlands; y.yu@uu.nl (Y.Y.); b.r.j.blokhuis@uu.nl (B.R.B.); j.garssen@uu.nl (J.G.); 2 Nutricia Research, 3584CT Utrecht, The Netherlands

**Keywords:** mast cells, colon cancer, 3D coculture, transcriptome

## Abstract

Mast cells (MCs) are one of the first immune cells recruited to a tumor. It is well recognized that MCs accumulate in colon cancer lesion and their density is associated with the clinical outcomes. However, the molecular mechanism of how colon cancer cells may modify MC function is still unclear. In this study, primary human MCs were generated from CD34^+^ progenitor cells and a 3D coculture model was developed to study the interplay between colon cancer cells and MCs. By comparing the transcriptomic profile of colon cancer-cocultured MCs versus control MCs, we identified a number of deregulated genes, such as MMP-2, VEGF-A, PDGF-A, COX2, NOTCH1 and ISG15, which contribute to the enrichment of cancer-related pathways. Intriguingly, pre-stimulation with a TLR2 agonist prior to colon cancer coculture induced upregulation of multiple interferon-inducible genes as well as MHC molecules in MCs. Our study provides an alternative approach to study the influence of colon cancer on MCs. The transcriptome signature of colon cancer-cocultured MCs may potentially reflect the mechanism of how colon cancer cells educate MCs to become pro-tumorigenic in the initial phase and how a subsequent inflammatory signal—e.g., TLR2 ligands—may modify their responses in the cancer milieu.

## 1. Introduction

Mast cells (MCs) are among the first immune cells recruited in the initial phase of tumorigenesis [1]. Once MCs migrate to a tumor site, many immunologic and non-immunologic factors present in the cancer microenvironment can regulate their phenotypes and functions [2,3]. It has been well recognized that MCs accumulate in colon cancer and their number is associated with microvessel formation and the disease prognosis [2,4]. The majority of studies show that MCs promote colon cancer progression [5,6,7,8,9,10], while only a few had an opposite observation and suggested that higher MC numbers correlate with better clinical outcomes [11,12]. Upon activation, MCs release a variety of mediators and therefore directly influence tumor growth or indirectly regulate the local immune responses [1,2]. For instance, angiogenic factors (e.g., VEGF) and growth factors (e.g., IL-8) facilitate angiogenesis and cancer cell proliferation [13,14,15,16]; matrix metalloproteinases (e.g., MMP-9) and proteases (tryptase and chymase) degrade extracellular matrix (ECM) components and thereby favor the implantation of tumor cells [17,18]. By releasing TGF-β, amphiregulin and adenosine, MCs can suppress the protective immune responses against cancer [18,19]. On the other hand, MCs can directly kill tumor cells through ROS (reactive oxygen species) and TNF-α [2,20]. They can also indirectly inhibit tumor growth through secreting heparin, IL-9 and stimulation of dendritic cell maturation [2,20]. Despite mounting evidence showing the impact of MC-derived mediators on tumor growth, the molecular mechanism is still unknown of how colon cancer cells modify MC phenotype and activity.

The current development of next-generation sequencing of RNA (RNA-seq) and Gene Ontology analysis [21] gives us an opportunity to look into the whole transcriptional landscape of cancer-associated MCs and to predict their activity. However, it is difficult to obtain sufficient numbers of MCs from human colon cancer and healthy colon tissues, due to the limited source of fresh human materials and technical challenges to isolate MCs from such tissue. In this study, primary MCs from human peripheral CD34^+^ stem cells were generated [22] and an in vitro 3D coculture model was developed to study the MC—colon cancer interaction. Our previous data have shown that human MCs promote colon cancer growth and this effect is enhanced by their cellular crosstalk [23]. To understand how colon cancer cells may modify MCs to be pro-tumorigenic, we compared the transcriptomic profile of colon cancer-cocultured MCs versus control MCs and sorted interesting genes using Ingenuity Pathway Analysis (IPA).

## 2. Results

### 2.1. Deregulated Genes in Colon Cancer-Cocultured MCs

**Figure 1:**
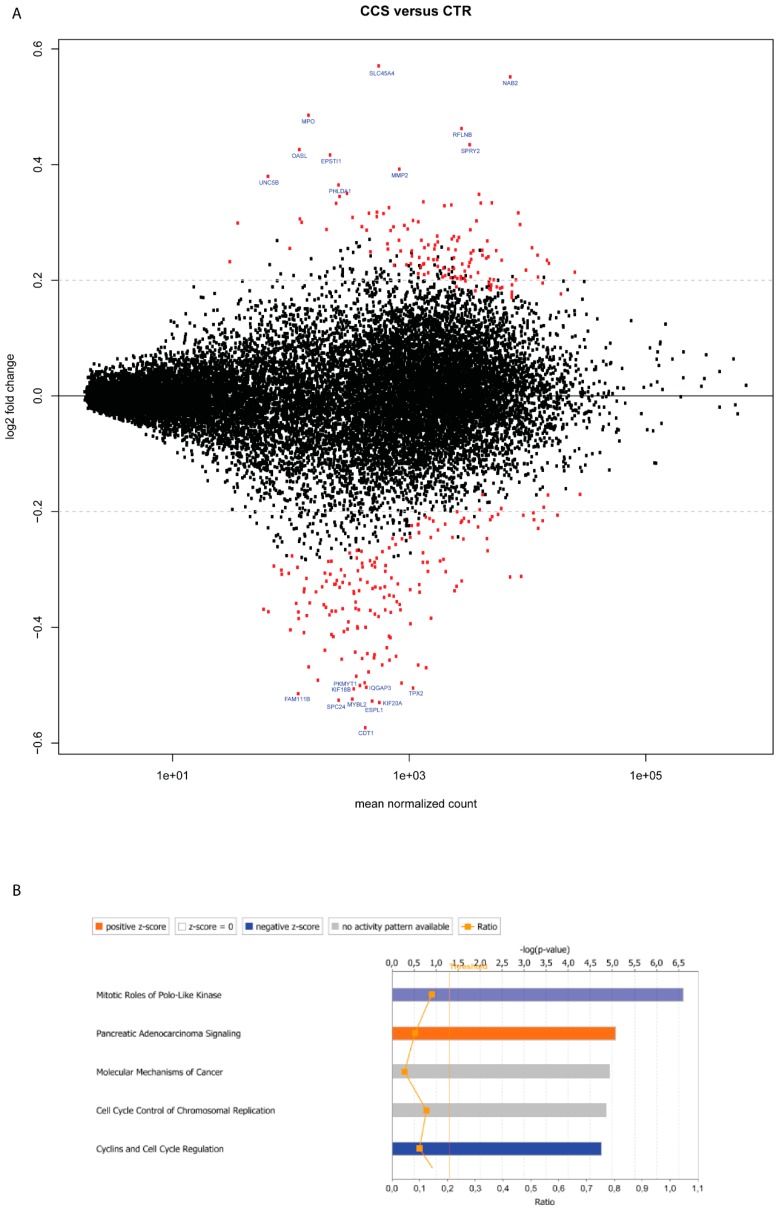
Deregulated genes in MCs cocultured with HT29 spheroids. (**A**) Plots of normalized mean count (gene expression) versus log_2_-transformed gene expression fold change (log_2_FC) for MCs cocultured with HT29 spheroids (CCS) versus MCs alone (CTR). Each dot represents a transcript. Dots in red were significantly differentially up- or downregulated (*p* < 0.05) (*n* = 2). Dots labeled with the gene symbol were the top 10 upregulated or downregulated genes. The *p* values were computed by the Wald test and corrected by the Benjamini-Hochberg procedure. The black horizontal line denotes the base line level and the grey horizontal line denotes the value of log_2_FC = 0.2. (**B**) The top 5 canonical pathways enriched in CCS. Pathways in red indicate activated and those in blue, suppressed. Pathways in grey indicate no functional prediction available. The vertical line denotes the significance level of α = 0.05. (-□-) The ratio represents the number of genes from the dataset that are part of the pathway.

To assess the influence of colon cancer cells on MC gene expression, we compared the transcriptome profile of MCs cocultured with HT29 spheroids (CCS) versus MCs cultured only in ECM without HT29 (CTR). Log_2_-transformed gene expression fold change (log_2_FC) in CCS was calculated over control CTR. At the specified significance level of corrected *p* value < 0.05 and log_2_FC > 0.2, deregulation of 281 genes was found in CCS (Figure 1A and Table 1). IPA pathway enrichment analysis showed the enhancement of “pancreatic adenocarcinoma signaling” and “molecular mechanism of cancer” (Figure 1B), which may support the significance of MCs in cancer pathology. Among the top 10 deregulated genes, MMP2 (Matrix Metallopeptidase 2) is well known for its role in colorectal cancer invasion [24] and EPSTI1 (Epithelial stromal interaction 1) and MPO (Myeloperoxidase) are related to tumor development [25,26].

On the other hand, pathways related to cell division, such as “mitotic roles of polo-like kinase” and “cyclins and cell cycle regulation” were suppressed (Figure 1B). Indeed, among the top 10 downregulated genes, KIF20A and KIF18B are linked to cell proliferation [27]. SPC24 and CDT1 regulate DNA replication [28] and ESPL1, MYBL2, TPX2 are related to cell mitosis/cell cycle control [29,30].

### 2.2. Gene Deregulation for Extracellular Mediators in Colon Cancer-Cocultured MCs

To more precisely understand how colon cancer cells may shape MCs to become pro-tumorigenic, we further sorted those 281 deregulated genes based on the following criteria: (1) MC markers; (2) involvement in colon cancer pathology or (3) involvement in tumor-stromal interaction. Genes for extracellular mediators were evaluated to estimate the action of MCs on colon cancer cells. Classical MC mediators, such as PDGF-A, VEGF-A, and MMP-2 were significantly upregulated in CCS (Table 2), suggesting that colon cancer cells potentially augment MC-mediated tumor growth, angiogenesis and invasion. Because non-protein mediators (e.g., amines, lipids) cannot be directly analyzed by RNA sequencing, key enzymes for their synthesis were evaluated. Intriguingly, PTGS2 (also known as COX2) was upregulated in CCS (Table 2). In addition to classic MC mediators, transcripts of TNFSF14 (also called LIGHT) and ISG15 (IFN-stimulated gene 15) were also upregulated in CCS (Table 2). LIGHT can be secreted by human MCs [31] and plays a role in colon cancer pathology [32,33]. ISG15 has been shown to enhance the tumorigenic potential of cancer stem cells [34]. Since ISG15 is also an interferon-stimulated gene, its upregulation may indicate the presence of interferon signals in the coculture. Indeed, another interferon inducible gene, i.e., EPSTI1, was also upregulated in CCS (Table 1).

### 2.3. Gene Deregulation for Membrane Receptors in Colon Cancer-Cocultured MCs

By examining the transcript for plasma membrane receptors in CCS, we may speculate the increased ligand-receptor interaction in the cellular crosstalk between MCs and HT29 cells. Human MCs constitutively express multiple PGE2 receptors (EP2, EP3 and EP4) [35]. Coculture with HT29 cells induced increased EP4 in MCs (Table 2), indicating a possible enhanced PGE2—EP4 interaction in the cellular crosstalk. Of note, NOTCH1 and its downstream molecule RELA (a nuclear factor NF-κB subunit) were upregulated in CCS (Table 2). In supporting this, HT29 cells secret high amounts of notch1 ligands (e.g., JAG2) [36]. Other receptors such as FZD1 (frizzled 1) and UNC5B (netrin receptor) were also upregulated in CCS (Table 2). Indeed, Wnt (ligand for frizzled-1) and Netrin can be secreted by colon cancer cells [37,38]. Upon stress, colon epithelial cells can release multiple alarmins, such as IL-18, IL-33 and ATP [39]. Based on this, we also observed upregulation of IL18RAP (an IL-1 receptor family) and P2RY11 (a purinergic receptor) in CCS (Table 2).

On the other hand, transcriptome analysis of membrane receptors may provide a better insight into how colon cancer cells may modify MC activity. For instance, ITGA3 (gene coding for α_3_β_1_ integrin), ITGA2 (gene coding for α_2_β_1_ integrin) as well as their downstream molecules, i.e., RHOB and CIT were found downregulated in CCS (Table 2). It has been shown that α_3_β_1_ integrin mediates MC adhesion and migration on basal membrane laminins [40] and α_2_β_1_ integrin is necessary for MC-induced acute inflammatory responses against bacteria [41]. These data, together with downregulated HMMR (hyaluronan mediated motility receptor) and TJP2 (tight junction protein zo-2) (Table 2), suggest a reduced cytokinesis, cytoskeleton organization and cell trafficking ability of CCS (Figure 1B).

### 2.4. Deregulated Genes in TLR2-Primed MCs Cocultured with Colon Cancer Cells

**Figure 2:**
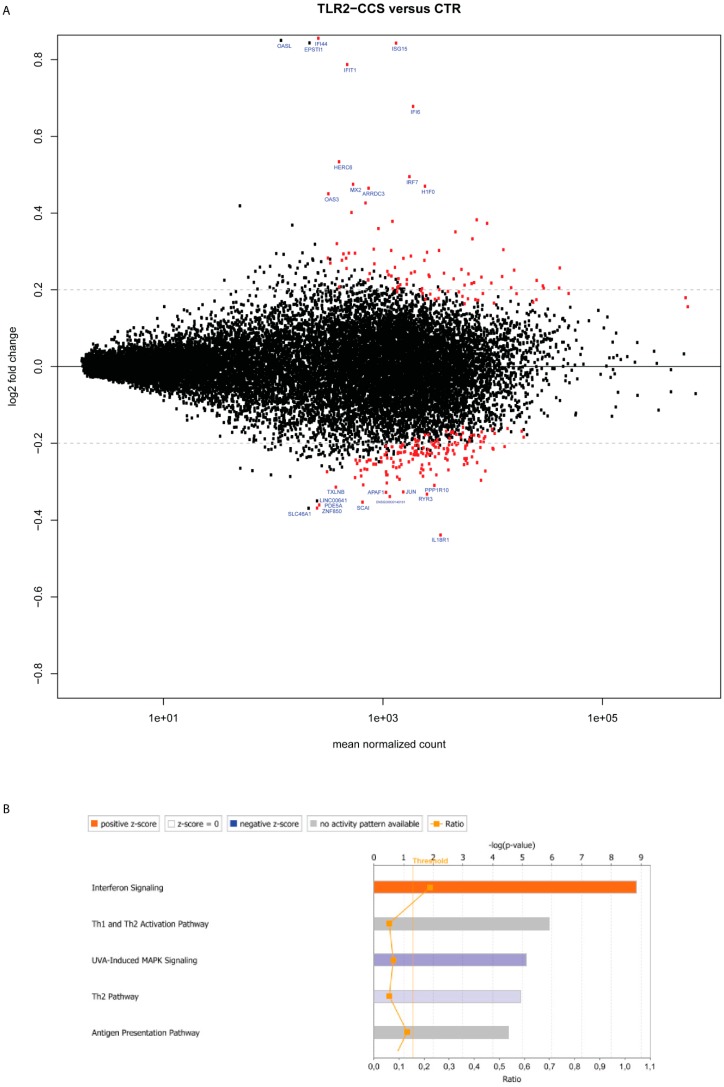
Deregulated genes in TLR2-primed MCs cocultured with HT29 spheroids. (**A**) Plots of normalized mean count (gene expression) versus log_2_-transformed gene expression fold change (log_2_FC) for TLR2-primed MCs cocultured with HT29 spheroids (TLR2-CCS) versus control MCs (CTR). Each dot represents a transcript. Dots in red were significantly differentially up- or downregulated (*p* < 0.05) (*n* = 2). Dots labeled with the gene symbol were the top 10 upregulated or downregulated genes. The *p* values were computed by the Wald test and were corrected by the Benjamini-Hochberg procedure. The black horizontal line denotes the base line level and the grey horizontal line denotes the value of log_2_FC = 0.2. (**B**) The top 5 canonical pathways enriched in TLR2-CCS. Pathways in red indicate activated and in blue suppressed. Pathways in grey indicate no functional prediction available. The vertical line denotes the significance level of α = 0.05. (-□-) The ratio represents the number of genes from the dataset that are part of this pathway.

We hypothesized that colon cancer cells may provide a first signal to stimulate MCs in the early phase, and as cancer grows, danger signals derived from stressed cancer cells or exogenous pathogens in the gut may give a second wave of stimuli. In our previous study, we showed that TLR2 pre-stimulation (FSL-1) of MCs can induce a stronger growth of HT29 spheroids [23]. To anticipate how the combination of HT29 and TLR2 stimuli may influence MC activity, we compared the transcriptome signature of MCs that received TLR2 stimulation prior to the coculture with HT29 spheroids (TLR2-CCS) versus control MCs (CTR). At the specified significance level of corrected *p* value < 0.05 and log_2_FC > 0.2, 197 genes were deregulated in TLR2-CCS (Figure 2A and Table 3). Pathway analysis showed the enhancement of “interferon signaling”, “antigen presenting pathway” and T helper cell pathways (Figure 2B). Among those 197 candidates, we further sorted the interesting candidates based on the previous selection criteria.

### 2.5. Gene Deregulation for Extracellular Mediators in TLR2-Primed MCs Cocultured with Colon Cancer Cells

Similar to CCS, increased MMP2, ISG15 and TNFSF14 were also found in TLR2-CCS (Table 4), suggesting that the upregulation might, most likely, be induced by HT29 cells instead of TLR2 stimulation. Of note, ISG15 was the most significant deregulated gene in TLR2-CCS with log_2_FC = 0.84 (Figure 2A and Table 3). This effect was not due to the direct consequence of TLR2 triggering, as no increase in ISG15 was observed in TLR2-primed MCs alone (data not shown). Moreover, upstream signaling molecules of ISG15, such as STAT1 and IRF7 (Interferon regulatory factor 7), were also found upregulated in TLR2-CCS (Table 4), supporting the enhancement of the “interferon signaling” pathway. In line with this, expression of other interferon inducible genes, i.e., IFI44, IFIT1, IFI6 and MX2, also significantly increased (Figure 2A and Table 3). Interestingly, gene coding for 5-lipoxygenase (ALOX5) was upregulated in TLR2-CCS (Table 4). Since 5-lipoxygenase is a key enzyme responsible for synthesizing leukotrienes in MCs, upregulation of ALOX5 may suggest an increased production of leukotrienes in TLR2-CCS. On the other hand, gene NDST2 (*n*-deacetylase/*n*-sulfotransferase-2) was downregulated in TLR2-CCS (Table 4) and NDST2 is a crucial enzyme for heparin biosynthesis in MCs. Different from CCS, there was no significant deregulation of VEGFA, PDGFA and COX2 in TLR2-CCS (data not shown).

### 2.6. Gene Deregulation for Membrane Receptors in TLR2-Primed MCs Cocultured with Colon Cancer Cells

Next, to determine to what extent the deregulated genes in TLR2-CCS are involved in antigen presenting signaling, we sorted genes that are enriched in this pathway. Intriguingly, MHC class molecules, such as HLA-B (MHC I, B), HLA-C (MHC I, C), HLA-DPA1 (MHC II DP a), HLA-DRA (MHC II DR a) and CD74 (MHC II γ chain), were significantly upregulated in TLR2-CCS (Table 4). Because this was not observed in TLR2-primed MCs alone (data not shown), we do not expect that TLR2 stimulation triggers the upregulation. In addition, ITGB7 (coding for ß7 integrin) was upregulated in TLR2-CCS (Table 4). ß7 integrin is an important molecule for MC homing to the small intestine in mice [42]. Nevertheless, there are no data available to predict its biological meaning in human MCs [43]. In contrast to CCS, no upregulation of ITGA2/3, EP4, NOTCH1, FZD1 or UNC5B was observed in TLR2-CCS (data not shown).

## 3. Discussion

Although a significant role for MCs in colon cancer is well recognized in mouse and human studies, the underlying mechanism is poorly defined. Here, we analyzed the effect of MC-colon cancer interplay on MC transcriptome profile. We identified a list of deregulated genes in MC when cocultured with HT29 colon cancer. Pathway enrichment analysis revealed these genes are involved in cancer-associated signaling. This study may initiate an understanding how colon cancer cells can shape the phenotype and function of MCs and vice versa, how MCs may contribute to colon cancer development.

**Figure 3:**
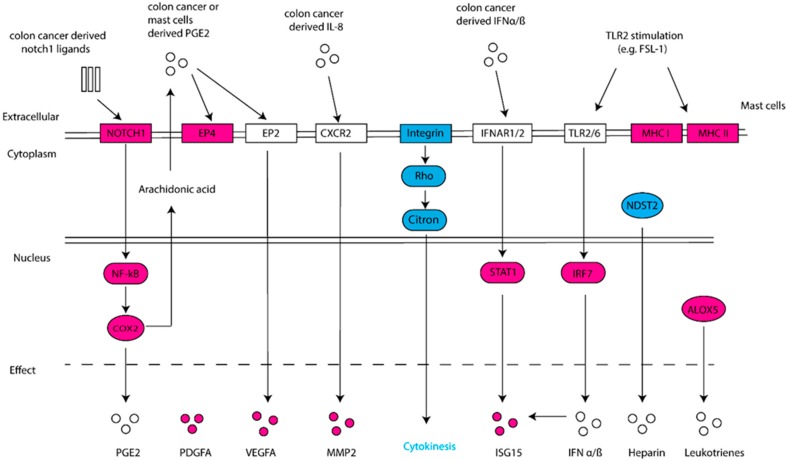
Schematic overview of deregulated genes and associated pathways. Differentially upregulated genes are shown in red and downregulated genes in blue. Pathways and associated upstream/downstream molecules are predicted.

We show that HT29 colon cancer cells induced an increased expression of VEGF-A, PDGF-A and MMP2 in MCs. This supports the notion that colon cancer can educate MCs to be a pro-tumorigenic player. It has been reported that IL-8 induces MMP2 production in endothelial and neuron cells [44,45]. Based on our previous finding, increased IL-8 levels were detected in the coculture [23]. We speculate that the upregulation of MMP2 in CCS might be, at least partly, caused by IL-8 derived from HT29. The upregulation of COX2 may indicate an increased activity of prostaglandin synthesis in CCS. Still, the production of prostaglandins is also dependent on the availability of substrate - arachidonic acid (AA), which is released from membrane lipids by phospholipase A2 (PLA2). In addition, prostaglandins play different roles in tumorigenesis. For instance, PGD2 has been shown to inhibit colitis-associated colon cancer [46], whereas PGE2 promotes tumor cell growth [47] and immunosuppression in the tumor microenvironment [48]. The upregulation of EP4 in CCS might support an increased interaction of EP4 with PGE2 in the coculture, where the latter can be secreted by colon cancer cells and/or MCs themselves. On the other hand, PGE2 can bind to EP2 on MCs, resulting in enhanced production of VEGF-A [49,50]. This speculation is in line with our observation that VEGF-A was upregulated in CCS as well as the coculture supernatant [23]. Pathway enrichment analysis showed the enhancement of “pancreatic adenocarcinoma signaling”, where the Notch1-NF-κB-COX2 pathway was activated (Figure 3). The inhibition of mitosis-related pathways may suggest a reduced proliferation of MCs when in contact with HT29. However, since the analysis was based on databases of mainly non-MC cells, experimental investigation is needed to confirm these pathways in human MCs.

Stressed and dying cancer cells release endogenous “danger” signals, such as ATP, S100 and HMGB1, which bind and activate the pattern recognition receptors (PRRs), frequently TLR2 or TLR4, to trigger immune responses [51]. As such, MCs were pre-stimulated with a TLR2 agonist (FSL-1) prior to the coculture with HT29 colon cancer. In particular, ISG15 was upregulated in CCS and most interestingly, its upregulation was enhanced upon TLR2 stimulation, leading to the most significant log_2_FC in TLR2-CCS. It has been shown that ISG15 can promote cancer stem cell growth [34], yet it still remains an open question whether it is a pro- or anti-tumor factor. Some studies suggest the effect depends on whether it is in a free or conjugated form [52]. For instance, free ISG15 can exert an antitumor response by activating the innate and adaptive immunity at the tumor site [53]. In addition to ISG15, other interferon inducible genes (e.g., IFI44, IFIT1, IFI6) were also upregulated in TLR2-CCS. Since type I IFNs are potent stimuli for ISG15 production as well as other interferon inducible genes [54], our data may imply that HT29 colon cancer could, through the secretion of IFNα/ß, increase ISG15 production in MCs (Figure 3).

Another interesting finding is the upregulation of MHC class molecules—i.e., MHC I B, MHC I C, HLA-DPA1, HLA-DRA and CD74—in TLR2-CCS. The notion that MCs function as antigen presenting cells is not new, since several studies have shown that IFN-γ combined with or without TLR agonists induces MHC II expression on MCs [55,56,57]. However, IFN-γ was not detectable in the coculture supernatant (data not shown), indicating that mediators other than IFN-γ are involved. On the other hand, IFN-α/ß has been shown to stimulate upregulation of MHC class molecules in dendritic cells (DCs) and IFN-α primed DCs proved to be more susceptible for TLR stimulation [58]. This leads to an interesting question whether IFN-α/ß together with a TLR2 agonist can induce MCs to become antigen-presenting cells. Since the MHC upregulation was not observed in MCs that only received TLR2 stimulation, we speculate that both the stimuli from HT29 coculture and TLR2 agonist are indispensable. Together, these data shed light on the potential of MCs being tailored to increase tumor immunogenicity.

Although most upregulated genes in CCS are associated with pro-tumor properties, the upregulation of TNFSF14 might have an opposite effect. Maker and co-workers have shown that increased expression of TNFSF14 enhances the activity of cytotoxic T-lymphocytes and thereby mediates immune eradication of colon cancer metastases [33,59]. Therefore, it is important to recognize the molecular profile of tumor infiltrating MCs, and the contribution of MCs may vary based on the balance of pro- and anti-tumor factors. This might, at least partly, explain why studies have opposite observations regarding the correlation of MC numbers and clinical outcomes in CRC. The limitation of our study is the small sample size (*n* = 2). As a consequence, some genes such as EPSTI1 and OASL display high log_2_FC in TLR2-CCS, but they did not reach statistical significance.

By using a 3D coculture model, our study provides an alternative approach to study the phenotype and activity of (colon) cancer-associated MCs. The transcriptome signature of HT29-cocultured MCs potentially reflects the mechanism of how colon cancer cells may shape MCs to become a pro-tumor player in the early phase and how a subsequent inflammatory signal (e.g., TLR2 ligands) may modify their responses in the cancer milieu. In addition, our data suggest the importance of lipid mediators in MC-colon cancer interaction. Nevertheless, the molecular signature as well as the downstream signaling are based on transcript expression and enrichment analysis. Future studies are needed to further validate the expression and function of targeted proteins and lipids produced by MCs in colon cancer.

## 4. Materials and Methods

Peripheral autologous hematopoietic stem cells derived from patients were used after written informed consent as approved by the ethics committee (TCBio 16-089) of the Utrecht Medical Center, Utrecht, the Netherlands, in accordance with the Declaration of Helsinki (59th WMA General Assembly, Seoul, October 2008), and in compliance with guidelines from the Ethical Committee and European Union legislation.

### 4.1. Generation of Primary Human MCs

CD34^+^-derived human MCs were generated from surplus autologous stem cell concentrates as previously described by Schmetzer at al. [22]. Briefly, frozen stem cell concentrates were rapidly thawed at 37 °C under sterile conditions and poured into a large cell culture flask (Greiner Bio-One, Kremsmünster, Austria). Then, 20% human serum albumin clinical solution (HSA) (Sanquin, Utrecht, the Netherlands), 6% hydroxyethyl starch clinical solution (Braun, Kronberg im Taunus, Germany) and RMPI containing 10 U/mL Heparin (LEO pharma, Ballerup, Denmark) were added slowly and consecutively to the cell concentrate. Cells were then filtered through a cell dissociation sieve (Sigma-Aldrich, St. Louis, MO, USA) and incubated with 200 U.I./mL DNAse (Roche, Basel, Switzerland) for 15 min. After washing, cells were resuspended in PBS containing 4% HSA and incubated with Fc-Block (Miltenyi Biotec, Bergisch Gladbach, Germany) for 15 min, CD34^+^ positive selection cocktail (STEMCELL Technologies, Vancouver, BC, Canada) for 15 min and nanoparticles for 10 min. Subsequently, CD34^+^ cells were sorted with an EasySep^®^ Magnet (STEMCELL Technologies, Vancouver, BC, Canada) according to the manufacturer’s protocol. Finally, sorted cells were resuspended in serum-free expansion medium (SFEM, STEMCELL Technologies) supplemented with human LDL (50 μg/mL, STEMCELL Technologies). On day 1, human recombinant IL-3 (100 ng/mL, Biolegend, San Diego, CA, USA) and SCF (100 ng/mL, Miltenyi Biotec) were added. Every three to four days, IL-3 and SCF were added to a final concentration of 20 ng/mL. At the end of the second week, MCs were maintained under 20 ng/mL SCF with the withdrawal of IL-3. From day 17 on, cells were used in described experiments. To obtain high quality, cell viability and purity, cells were further purified using a dead cell removal kit (Miltenyi Biotec) were added. Every three to four days, IL-3 and SCF were added to a final concentration of 20 ng/mL) followed by CD117 positive selection (Miltenyi Biotec) according to the manufacturer’s protocol. Mature MCs were identified based on the surface expression of CD117 and FcεRIa by flow cytometry, as previously described [23].

### 4.2. Colon Cancer Cell Lines

Human colon cancer cells HT29 were obtained from American Type Tissue Culture Collection. HT29 was grown in McCoy medium (Thermo Fisher Scientific, Waltham, MA, USA) supplemented with heat-inactivated 10% fetal calf serum (FCS, Thermo Fisher Scientific), and 100 μg/mL penicillin and streptomycin (Thermo Fisher Scientific). Cells were cultured in a humidified 37 °C/5% CO_2_ incubator.

### 4.3. Colon Cancer Spheroids

**Figure 4:**
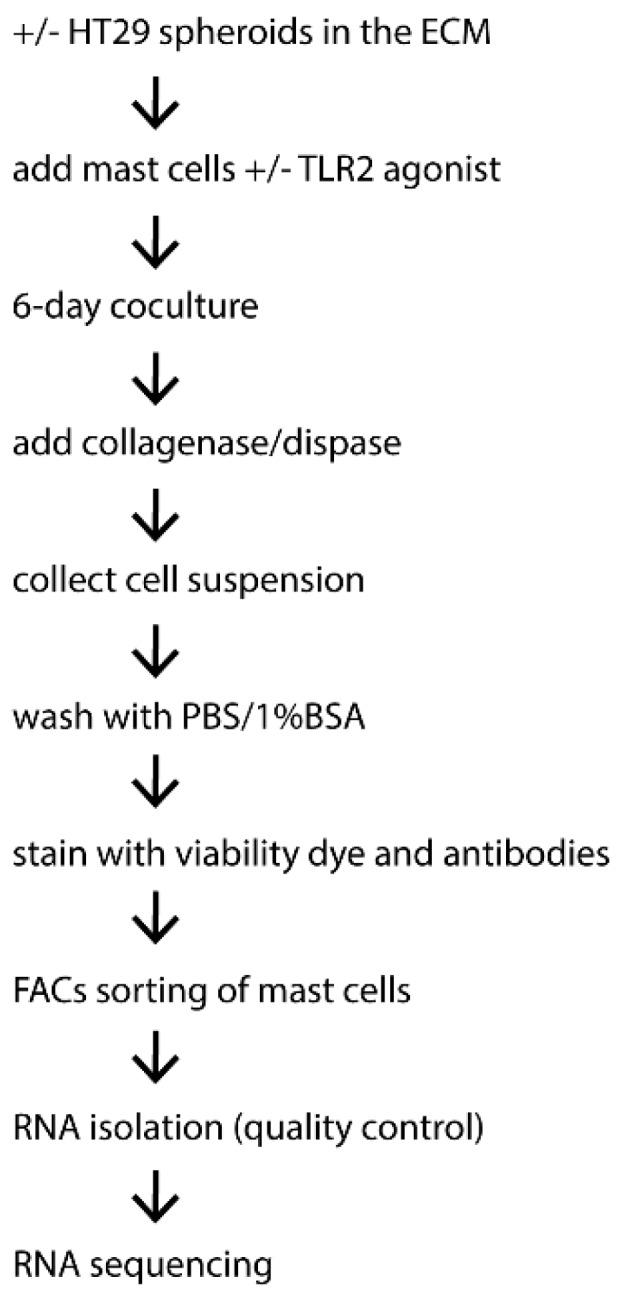
Brief overview of the experimental procedure to obtain mast cell RNA samples from the coculture with colon cancer spheroids.

Multicellular cancer spheroids were formed using the hanging drop method [60]. Briefly, drops of 250 HT29 cells in 20 µL were made in standard medium and incubated upside-down for 4 days. Cancer spheroids were then embedded in extracellular matrix (ECM) as previously described [61]. ECM mixture was prepared on ice containing Matrigel (Corning Inc., Corning, NY, USA) and non-pepsinized rat-tail collagen type I (2.3 mg/mL) (Corning Inc.) at a ratio of 1:1 or 1:100. Spheroids suspended in 40 µL of pre-heated culture medium were mixed with 200 µL of 1:1 ECM (Matrigel: Collagen type I). 50 µL drops of spheroid-ECM mixtures were then placed in a 24-well plate previously coated with 1:100 ECM (Matrigel: Collagen type I) base layer. For full polymerization of the gel, plates were incubated at 37 °C for 1 h. Subsequently, 1 mL coculture medium with or without human MCs (2 × 10^5^/mL) was added. Alternately, MCs were pre-stimulated with FSL-1 (1 µg/mL, InvivoGen, San Diego, CA, USA) at 37 °C for 4 h and thereafter extensively washed to remove excess FSL-1. After 6-day coculture, MCs were isolated from the coculture to obtain RNA samples (Figure 4).

### 4.4. Isolation and Purification of MCs

Collagenase/dispase (1 mg/mL) (Sigma-Aldrich) was added to the ECM mixture and incubated at 37 °C for 1 h. Cells were then washed with PBS supplemented with 1% BSA and subsequently stained with viability dye YO-PRO1 and antibodies (Thermo Fisher Scientific) for CD117 and FcεRIa. Viable cells were gated based on negative expression of YO-PRO1, followed by the sorting based on co-expression of FcεRIa and CD117 using BD Influx™ cell sorter (BD Biosciences, San Jose, CA, USA). Control MCs cultured in ECM without HT29 spheroid were isolated and sorted in an identical way as HT29-cocultured MCs. The purity of isolated MCs was identified by flow cytometry as previously reported [23]. Around 4.5 × 10^4^ and 3 × 10^5^ purified MCs were obtained from donor 1 and 2, respectively, for subsequent RNA isolation. RNA was isolated using PureLink RNA mini kit (Thermo Fisher Scientific), according to the manufacturer’s protocol.

### 4.5. Transcriptome Analysis

RNA samples of human MCs were sequenced on an Illumina Nextseq500 platform according to the manufacturer’s procedure by the Utrecht Sequencing Facility of the Utrecht University (http://www.useq.nl/) (access on 16 May 2018). Sequencing libraries were generated using TruSeq Stranded mRNA poly A kit. Sequence reads were checked for quality by FastQC (v0.11.4) after which reads were aligned to GRCh37 using STAR (v2.4.2a) and read groups were added using Picard (v1.141). All samples passed QC and were subsequently processed using HTSeq-count (v0.6.1) on ENSEMBL gene definitions (GRCh37, release 74).

### 4.6. Detecting Differential Gene Expression

R statistical software was used to identify deregulated genes. The *p* values were computed by the Wald test and corrected by the Benjamini-Hochberg procedure. Transcripts with log_2_-transformed gene expression fold change (log_2_FC) > 0.2 and adjusted *p* value < 0.05 were deemed differentially expressed. Enriched canonical pathways and MC marker genes were analyzed using IPA^®^ software (QIAGEN, Hilden, Germany) based on the cutoff log_2_FC > 0.2 and corrected *p* value < 0.05.

## Figures and Tables

**Table 1:** The top 10 deregulated genes in CCS versus CTR.

Gene Symbol	Name	Gene Expression ^‡^	Log_2_FC	Adjusted *p* Value
upregulated				
SLC45A4	Solute carrier family 45 member 4	1.07	0.57	****
NAB2	Nuclear polyadenylated RNA-binding protein	40.76	0.55	****
MPO	Myeloperoxidase	0.49	0.49	****
RFLNB	Refilin-B	18.00	0.46	****
SPRY2	Protein sprouty homolog 2	19.00	0.43	****
OASL	2′-5′-oligoadenylate synthase-like protein	0.21	0.43	****
EPSTI1	Epithelial-stromal interaction protein 1	0.39	0.42	****
MMP2	Matrix metalloproteinase-2	3.23	0.39	****
UNC5B	Netrin receptor UNC5B	0.20	0.38	****
PHLDA1	Pleckstrin homology-like domain family A member 1	0.54	0.36	****
downregulated				
CDT1	DNA replication factor	3.34	−0.57	****
KIF20A	Kinesin-like protein KIF20A	3.54	−0.53	****
ESPL1	Separin	1.27	−0.53	****
SPC24	Kinetochore protein Spc24	2.20	−0.53	****
MYBL2	Myb-related protein B	2.33	−0.52	****
KIF18B	Kinesin-like protein KIF18B	1.66	−0.51	****
FAM111B	Protein FAM111B	0.68	−0.51	****
TPX2	Targeting protein for Xklp2	7.41	−0.50	****
IQGAP3	Ras GTPase-activating-like protein IQGAP3	1.59	−0.50	****
PKMYT1	Membrane-associated tyrosine- and threonine-specific cdc2-inhibitory kinase	1.88	−0.50	****

^‡^ RPKM (Reads Per Kilobase Million) of control MCs. **** *p* ≤ 0.0001.

**Table 2:** Genes of interest in CCS versus CTR.

Gene Symbol	Name	Cytolocation	Gene Expression *	Log_2_FC	Adjusted *p* Value
MMP2	Matrix metalloproteinase-2	Extracellular	3.23	0.39	****
VEGFA	Vascular endothelial growth factor A	Extracellular	2.51	0.23	*
PDGFA	Platelet-derived growth factor subunit A	Extracellular	3.84	0.29	**
TNFSF14	Tumor necrosis factor ligand superfamily member 14	Extracellular	2.04	0.31	**
ISG15	Interferon-stimulated gene 15	Extracellular	25.01	0.34	**
NPTX1	Neuronal pentraxin-1	Extracellular	1.66	−0.38	***
PTGS2	Prostaglandin G/H synthase 2	Cytoplasm	1.42	0.35	**
RHOB	Rho-related GTP-binding protein RhoB	Cytoplasm	35.05	−0.22	*
CIT	Citron Rho-interacting kinase	Cytoplasm	1.32	−0.37	***
RELA	Nuclear factor NF-kappa-B p65 subunit	Nucleus	20.11	0.24	*
STAT1	Signal transducer and activator of transcription 1-alpha/beta	Nucleus	14.78	0.27	**
NOTCH1	Neurogenic locus notch homolog protein 1	Membrane	10.18	0.35	****
PTGER4	Prostaglandin E2 receptor EP4 subtype	Membrane	1.16	0.29	*
FZD1	Frizzled-1	Membrane	3.09	0.32	*
UNC5B	Netrin receptor UNC5B	Membrane	0.20	0.38	***
IL18RAP	Interleukin-18 receptor accessory protein	Membrane	0.56	0.25	*
P2RY11	P2Y purinoceptor 11	Membrane	2.06	0.33	**
ITGA3	Integrin alpha-3	Membrane	14.56	−0.21	**
ITGA2	Integrin alpha-2	Membrane	1.19	−0.27	*
HMMR	Hyaluronan mediated motility receptor	Membrane	2.19	−0.44	****
TJP2	Tight junction protein ZO-2	Membrane	11.75	−0.22	*

^‡^ RPKM (Reads Per Kilobase Million) of control MCs. * *p* ≤ 0.05; ** *p* ≤ 0.01; *p* ≤ 0.001; **** *p* ≤ 0.0001.

**Table 3:** The top-10 deregulated genes in TLR2-CCS versus CTR.

Gene Symbol	Name	Gene Expression ^‡^	Log_2_FC	Adjusted *p* Value
upregulated				
IFI44	Interferon-induced protein 44	0.90	0.86	****
ISG15	Interferon-stimulated gene 15	25.01	0.84	****
IFIT1	Interferon-induced protein with tetratricopeptide repeats 1	2.05	0.79	****
IFI6	Interferon alpha-inducible protein 6	14.49	0.68	****
HERC6	Probable E3 ubiquitin-protein ligase HERC6	1.24	0.53	****
IRF7	Interferon regulatory factor 7	13.80	0.49	****
H1F0	Histone H1.0	21.75	0.47	****
MX2	Interferon-induced GTP-binding protein Mx2	0.79	0.47	****
ARRDC3	Arrestin domain-containing protein 3	3.42	0.46	****
OAS3	2′-5′-oligoadenylate synthase 3	0.07	0.45	****
downregulated				
IL18R1	Interleukin-18 receptor 1	26.24	−0.44	****
ZNF850	Zinc finger protein 850	1.10	−0.37	**
PDE5A	cGMP-specific 3′,5′-cyclic phosphodiesterase	0.98	−0.36	**
SCAI	Protein SCAI	1.75	−0.35	***
ENSG00000140181	N/A	5.13	−0.34	***
APAF1	Apoptotic protease-activating factor 1	4.32	−0.33	**
JUN	Transcription factor AP-1	16.81	−0.33	**
RYR3	Ryanodine receptor 3	5.47	−0.33	****
PPP1R10	Serine/threonine-protein phosphatase 1 regulatory subunit 10	17.85	−0.31	****
TXLNB	Beta-taxilin	2.86	−0.31	*

^‡^ RPKM (Reads Per Kilobase Million) of control MCs. * *p* ≤ 0.05; ** *p* ≤ 0.01; *p* ≤ 0.001; **** *p* ≤ 0.0001.

**Table 4:** Genes of interest in TLR2-CCS versus CTR.

Gene Symbol	Name	Cytolocation	Gene Expression *	Log_2_FC	Adjusted *p* Value
ISG15	Interferon-stimulated gene 15	Extracellular	25.01	0.84	****
MMP2	Matrix metalloproteinase-2	Extracellular	3.23	0.31	**
TNFSF14	Tumor necrosis factor ligand superfamily member 14	Extracellular	2.04	0.27	*
NDST2	Bifunctional heparan sulfate N-deacetylase/N-sulfotransferase 2	Cytoplasm	8.66	−0.21	*
ALOX5	Arachidonate 5-lipoxygenase	Nucleus	35.70	0.21	*
IRF7	Interferon regulatory factor 7	Nucleus	13.79	0.49	****
STAT1	Signal transducer and activator of transcription 1-alpha/beta	Nucleus	14.78	0.35	****
HLA-B	HLA class I histocompatibility antigen, B-51 alpha chain	Membrane	127.04	0.25	**
HLA-C	HLA class I histocompatibility antigen, Cw-12 alpha chain	Membrane	148.74	0.21	*
HLA-DPA1	HLA class II histocompatibility antigen, DP alpha 1 chain	Membrane	6.69	0.21	*
HLA-DRA	HLA class II histocompatibility antigen, DR alpha chain	Membrane	4.57	0.28	*
CD74	HLA class II histocompatibility antigen gamma chain	Membrane	17.18	0.30	***
ITGB7	Integrin beta-7	Membrane	7.11	0.23	*

^‡^ RPKM (Reads Per Kilobase Million) of control MCs. * *p* ≤ 0.05; ** *p* ≤ 0.01; *p* ≤ 0.001; **** *p* ≤ 0.0001.
